# Hyperoside promotes pollen tube growth by regulating the depolymerization effect of actin-depolymerizing factor 1 on microfilaments in okra

**DOI:** 10.1038/s41438-021-00578-z

**Published:** 2021-07-01

**Authors:** Biying Dong, Qing Yang, Zhihua Song, Lili Niu, Hongyan Cao, Tengyue Liu, Tingting Du, Wanlong Yang, Meng Qi, Ting Chen, Mengying Wang, Haojie Jin, Dong Meng, Yujie Fu

**Affiliations:** 1 Beijing Advanced Innovation Center for Tree Breeding by Molecular Design, College of Forestry, Beijing Forestry University, Beijing, 100000 China; 2 Key Laboratory of Forest Plant Ecology, Ministry of Education, Northeast Forestry University, Harbin, 150000 China

**Keywords:** Plant molecular biology, Plant breeding

## Abstract

Mature pollen germinates rapidly on the stigma, extending its pollen tube to deliver sperm cells to the ovule for fertilization. The success of this process is an important factor that limits output. The flavonoid content increased significantly during pollen germination and pollen tube growth, which suggests it may play an important role in these processes. However, the specific mechanism of this involvement has been little researched. Our previous research found that hyperoside can prolong the flowering period of *Abelmoschus esculentus* (okra), but its specific mechanism is still unclear. Therefore, in this study, we focused on the effect of hyperoside in regulating the actin-depolymerizing factor (ADF), which further affects the germination and growth of pollen. We found that hyperoside can prolong the effective pollination period of okra by 2–3-fold and promote the growth of pollen tubes in the style. Then, we used *Nicotiana benthamiana* cells as a research system and found that hyperoside accelerates the depolymerization of intercellular microfilaments. Hyperoside can promote pollen germination and pollen tube elongation in vitro. Moreover, *AeADF1* was identified out of all *AeADF* genes as being highly expressed in pollen tubes in response to hyperoside. In addition, hyperoside promoted AeADF1-mediated microfilament dissipation according to microfilament severing experiments in vitro. In the pollen tube, the gene expression of *AeADF1* was reduced to 1/5 by oligonucleotide transfection. The decrease in the expression level of *AeADF1* partially reduced the promoting effect of hyperoside on pollen germination and pollen tube growth. This research provides new research directions for flavonoids in reproductive development.

## Introduction

In a suitable environment, the pollen on the stigma germinates and grows pollen tubes; then, the pollen tube extends toward the ovule. As pollen tubes pass rapidly through the style, sperm cells are transferred to the ovule for fertilization^1^. Flavonoids play a significant role in the growth of pollen tubes^2^. Chalcone synthase (CHS), as a key enzyme in the flavonoid synthesis pathway, plays an important role in the synthesis of flavonoids. A study on CHS mutants of maize and petunia showed that pollen deficient in flavonoids failed to produce functioning pollen tubes. By applying specific flavonols to pollen or the stigma during pollination, the defect can be overcome, and fertility can be restored^3,4^. The *Arabidopsis* CHS mutant (*tt4*) also showed reduced seed setting and reduced pollen germination in vitro^5^. These reports indicate that flavonoids may interfere with pollen germination and pollen tube growth. However, the specific mechanism by which flavonoids affect pollen germination and pollen tube growth is unclear. Hyperoside, also known as quercetin-3-O-β-D-galactopyranoside, is a flavonol glycoside compound^6^. Our previous research showed that the content of hyperoside in *Abelmoschus esculentus* (okra) highly accumulates during flowering, as it is a signal substance that affects the length of the flowering period^6,7^.

For some plants, such as *Epiphyllum oxypetalum*, *Opuntia stricta*, and *A. esculentus*, the flowering period can be maintained for only a few hours. Such flowers often possess high ornamental and medicinal value. The petals and seeds of *A. esculentus*, a medicinal herb of the Malvaceae (mallow) family, have edible and medicinal compounds^8^. Medicinal ingredients are abundant in *A. esculentus*, especially in the petals^9^, and the concentration of active compounds in the petals peaks before they begin to wilt. Although most studies have focused on separating and extracting medicinal ingredients from *A. esculentus* flowers, it would be valuable to prolong the flowering period and improve the efficiency of fertilization during a short period of time^10–12^. Hyperoside is a major pharmacologically active component in *A. esculentus*. Many studies have already shown that hyperoside has pharmacological effects, such as relieving oxidative stress injury in cells and anticancer properties^13–15^.

Pollen tube growth is an important part of the fertilization process, and the actin cytoskeleton plays a critical role in pollen tube growth by supporting organelle movement^16^. The actin cytoskeleton refers to a protein fiber network framework composed of microtubules, microfilaments, and intermediate filaments. Microfilaments, which are called filamentous actin (F-actin), are composed of multimers of globular actin (G-actin) monomers^17^. In addition to actin fibers, there are many microfilament-binding proteins involved in the microfilament system. These microfilament-binding proteins are involved in the formation of high-level microfilament fibers, regulate the dynamic assembly of actin fibers, and perform specific functions^18–20^. Actin dynamics (i.e., assembly and disassembly) exhibit circadian regulation during pollen tube development^21,22^. Actin-depolymerizing factor (ADF) is one of the most widely studied proteins and can combine with monomeric or fibrous actin to accelerate the decomposition of actin subunits. There are 11 *ADF* genes in Arabidopsis, and they exhibit opposing biochemical properties. *AtADF1* belongs to the class I ADFs and has an F-actin depolymerization function; a high concentration of ADF protein has a higher depolymerization ability. In contrast, *AtADF5* belongs to the class III ADFs and has an F-actin-binding function^23–25^.

In this research, we started from the phenomenon that hyperoside has a positive regulatory effect on pollen tube growth and found that hyperoside promotes the depolymerization of microfilaments in an *Nicotiana benthamiana* cell system. Furthermore, we found that *AeADF1* is highly expressed in pollen in response to hyperoside and plays a significant role in pollen germination and pollen tube growth by severing actin. This research revealed that hyperoside promotes the severing efficiency of AeADF1 protein on microfilaments to promote pollen germination and pollen tube growth. This research provides new research directions for exploring the mechanism of flavonoids in other plants during flower development.

## Results

### Hyperoside increased cleavage rate of microfilaments in the plant cell

**Fig. 1:**
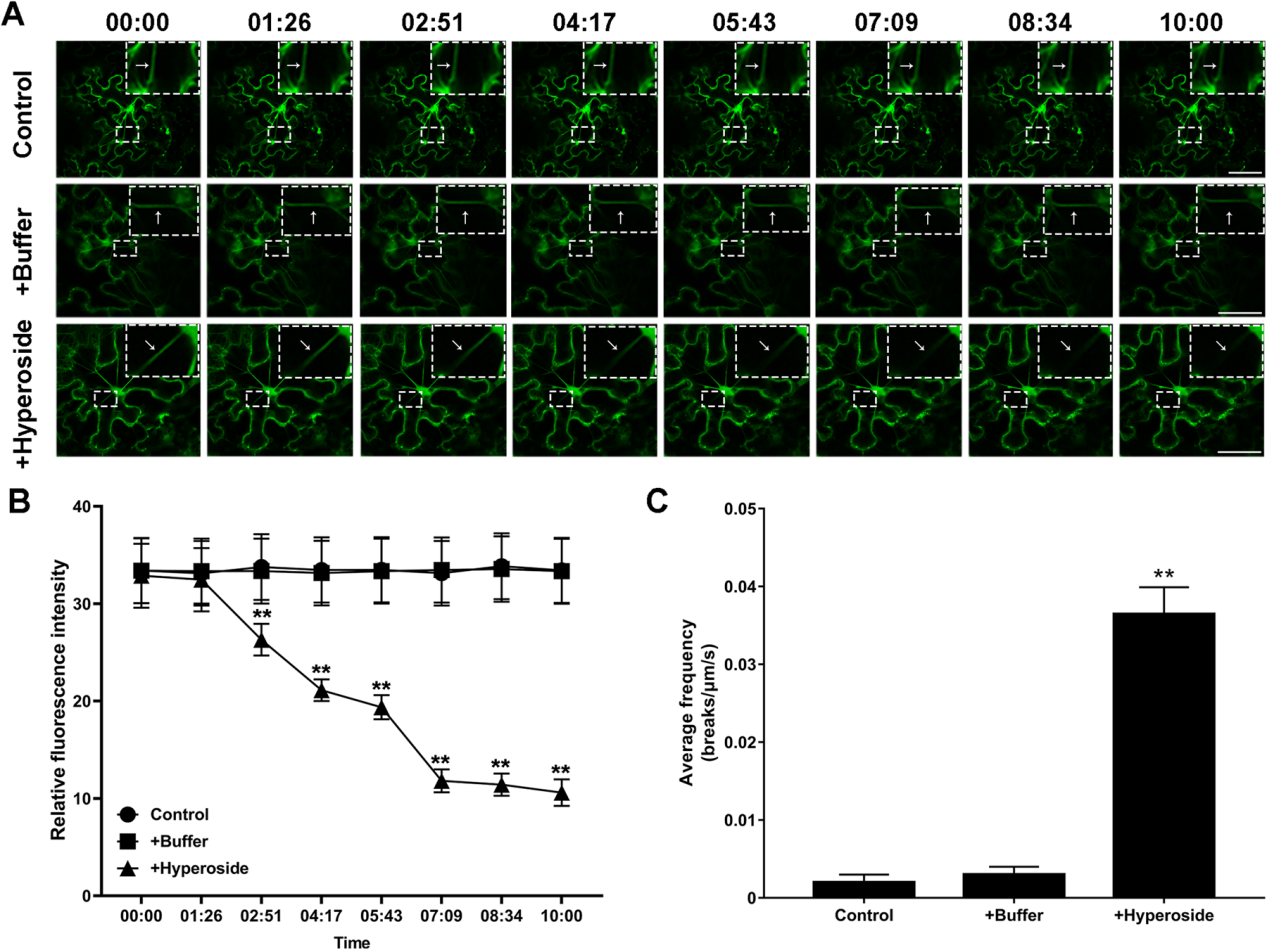
Hyperoside promoted the depolymerization of microfilaments in *N. benthamiana* cells. **A** Control and treatment with buffer and hyperoside (0.1 mM) in the leaves of *N. benthamiana* transiently expressing Lifeact-GFP. The position indicated by the white arrow is an enlarged microfilament. Bars = 50 μm. **B** The relative fluorescence intensity inside the white rectangle in (**A**). **C** The average frequency under different treatments in (**A**). Each treatment had three biological replicates, and error bars display the standard error of the sample. **P < 0.01 (Student’s *t* test)

We constructed a fusion expression vector of *Lifeact* and *eGFP* and then expressed them in *N. benthamiana* cells by transient transfection technology. The morphology of microfilaments in *N. benthamiana* cells sprayed with buffer solution only, sprayed with buffer solution containing 0.1 mM hyperoside, and not sprayed with any solution was examined under a laser confocal microscope. The results showed that the morphology of the microfilaments in the *N. benthamiana* cells sprayed with buffer solution was consistent with that of the control, and the microfilaments were not depolymerized within 10 min. In the *N. benthamiana* cells sprayed with hyperoside, an obvious process of microfilament depolymerization was observed. The depolymerization effect of the microfilaments in the cells had already been observed at 4 min and 17 s post hyperoside spraying (Fig. 1A, Supplemental Movies 1–3). To further quantify this process, we measured the relative fluorescence intensity in a similarly sized area in the control and both treatments and calculated the depolymerization time of the microfilaments per micrometer to indicate the depolymerization speed of the microfilaments (Fig. 1B, C). These results are consistent with our observations that hyperoside accelerates the depolymerization rate of cell filaments.

### Hyperoside prolongs the effective pollination period of okra

**Fig. 2:**
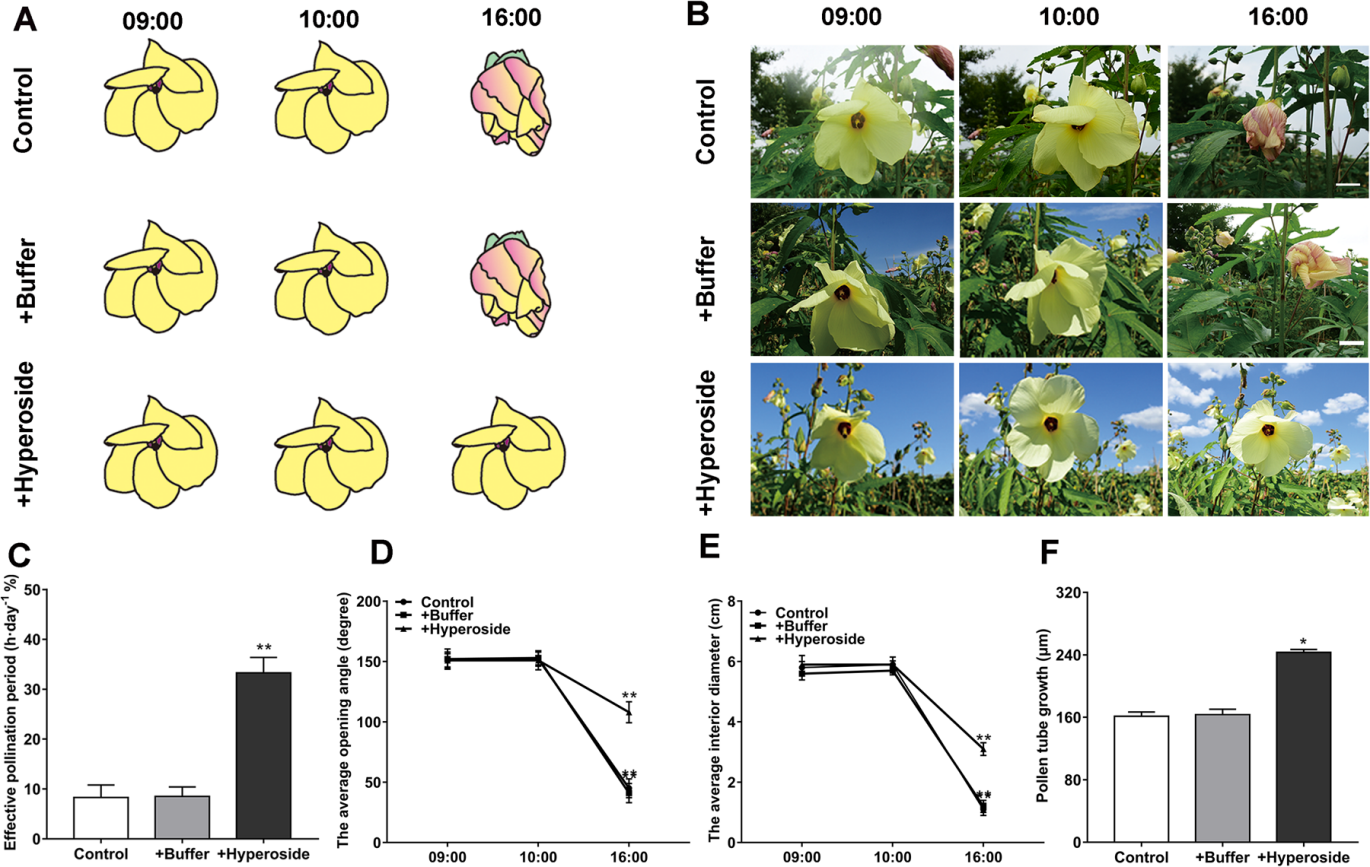
Hyperoside prolonged the flowering period and increased the effective pollination rate and pollen tube growth. **A** Schematic chart of the prolonged effective pollination period after adding hyperoside. **B** Time-lapse images of a flower from an untreated plant, a flower from a plant treated with buffer solution only, and a flower from a plant treated with a buffer solution containing 50 mg/l hyperoside. The processed photos of each group were taken on the same day. The scale bars are 1.5 cm. **C** The effective pollination period was analyzed in the control, buffer addition, and hyperoside addition groups. **D**, **E** The average interior diameter and opening angle of the flowers were analyzed at 9:00, 10:00. and 16:00. The changes in total flavonoids were also measured in the three groups. **F** The average length of pollen tubes in the style of flowers from control plants and plants after spraying buffer or hyperoside for 40 min. In **C**–**F**, each treatment had three biological replicates, and error bars display the standard error of the sample. *P < 0.05, **P < 0.01 (Student’s *t* test)

Since the depolymerization of filaments in cells is closely related to flower development^26^, we further explored whether hyperoside can affect flower development. As the effects of hyperoside on different plants may have certain differences, we did not verify them on *N. benthamiana*. We chose okra, which has only a one-day flowering period, as our experimental object to facilitate our experimental observation. In this study, we found that the exogenous application of hyperoside can prolong the effective pollination period of *A. esculentus*, with a flowering period from 9:00 to 16:00, or approximately 7 h (Fig. 2A–C). To describe the prolongation of the effective pollination period in detail, the average opening angle and the average interior diameter of flowers during the flowering period were measured. Compared with the control and buffer groups, the average opening angle and the average interior diameter of flowers increased continuously for ~3 h after spraying hyperoside (Fig. 2D, E). The length of the effective pollination period is an important factor affecting plant reproduction, as is the elongation of pollen tubes. Therefore, we further tested whether hyperoside has an effect on the elongation of pollen tubes. *A. esculentus* is a typical self-pollinated plant in which self-pollen is used to pollinate the pistils. We observed the length of the pollen tube in the style with aniline blue staining. The pollen tube length in the control only reached 70% of the tube length in the hyperoside treatment group. The pollen tube growth of the pollen treated with the control and buffer solutions was significantly slower than that treated with hyperoside (Fig. 2F). These results indicated that hyperoside treatment can positively affect both the length of the effective pollination period and pollen tube growth.

### Among the genes encoding actin-binding proteins, *AeADF1* and *AeADF5* respond most strongly to hyperoside

**Fig. 3:**
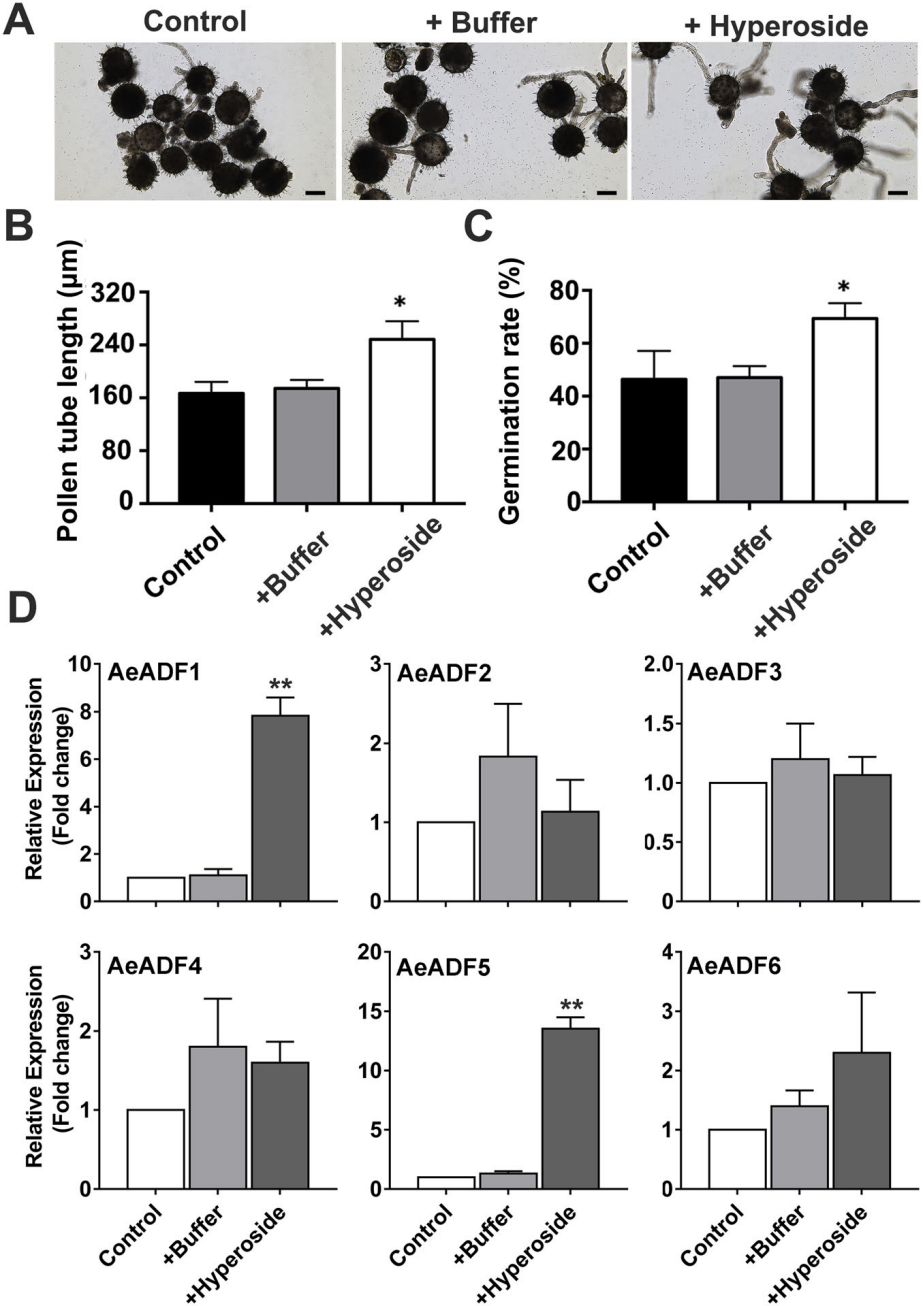
Screening of ADF genes in pollen that respond to hyperoside. **A** Pollen tube germination in the control and treatment with buffer and hyperoside (0.1 mM) groups. The length of the pollen tube (**B**) and germination rate (**C**) when treated with buffer and hyperoside for 2 h or not treated. Each treatment had three biological replicates, and error bars display the standard error of the sample. *P < 0.05, **P < 0.01 (Student’s *t* test). **D** The gene expression of *AeADF1–6* after treatment with buffer and hyperoside (0.1 mM) for 2 h or after no treatment

Exogenous application of hyperoside can prolong the effective pollination period of okra, and the germination and elongation of the pollen tube during the effective pollination period are the most important factors affecting pollination. Therefore, we further observed whether hyperoside has a certain effect on the elongation of pollen tubes in vitro. To determine whether hyperoside affects pollen germination and pollen tube growth, we measured the pollen germination rate and the average length of pollen tubes in vitro. In the pollen germination test, we found that the germination rate of untreated and buffer-treated pollen grains was significantly lower than that of hyperoside-treated pollen grains (Fig. 3A–C).

To complete fertilization, the pollen tube must enter the ovule. Whether actin filaments and actin-binding proteins play a significant role in the polar growth of pollen tubes is a popular research topic^27,28^. Since the *A. esculentus* genome has not been sequenced, we first designed degenerate primers to clone the full-length sequences of the 6 *ADF* genes and then designed specific primers to detect the transcript level. To determine which *AeADF* gene responded the most, we first cloned all six *AeADF* genes in flowers. In total, 6 full-length *AeADF* genes were obtained, and the transcript levels of *AeADF1* and *AeADF5* were upregulated the most, up to tenfold, after spraying with hyperoside (Fig. 3D).

### Isolation and bioinformatic analysis of AeADF1

**Fig. 4:**
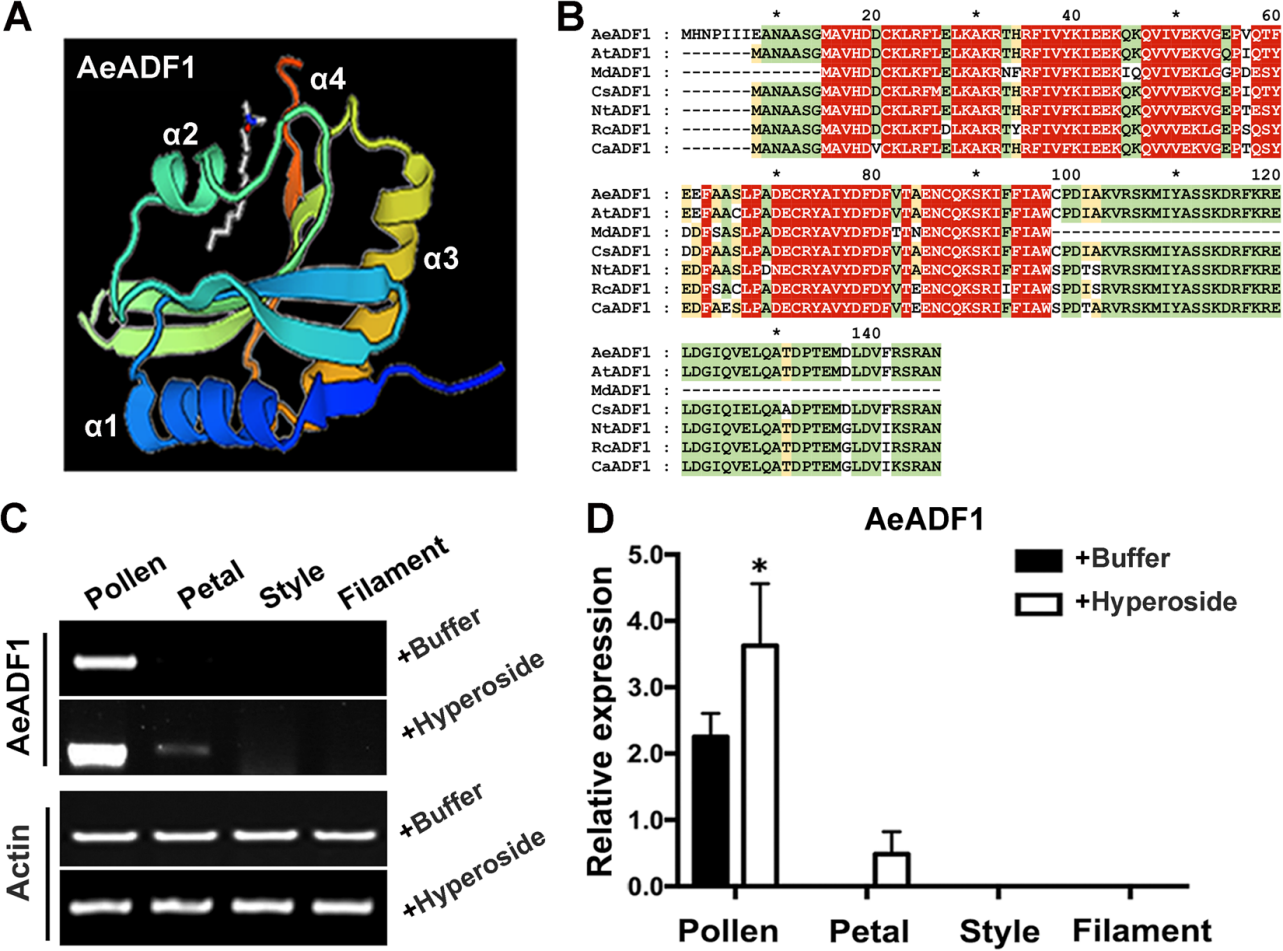
Analysis of the basic properties of AeADF1. **A** The homology model was created using the Phyre2 website (www.sbg.bio.ic.ac.uk/phyre2). The structures of AeADF1 and homologous ADF1 proteins were predicted with a >90% confidence interval. **B** Conserved protein domains of AeADF1 and homologous ADF1 proteins were predicted using the SMART database (http://smart.embl-heidelberg.de/) to confirm that they are members of the ADF family. **C** Semiquantitative reverse transcription-PCR analysis of *AeADF1* in the pollen, petals, styles, and filaments of control and hyperoside-treated flowers. *A. esculentus* actin was used as a control. **D** qRT-PCR analysis of *AeADF1* expression in the pollen, petals, styles, and filaments of control and hyperoside-treated flowers. Each treatment had three biological replicates, and error bars display the standard error of the sample. *P < 0.05 (Student’s *t* test)

By comparing the protein sequences of AeADF1, AeADF5, and AtADF1, it was found that the alpha helix motifs present in AtADF1 that play a significant role in the function of severing microfilaments existed in the AeADF1 protein, while the structures of the AeADF5 and AtADF1 proteins were quite different. At the same time, studies by Dong et al. showed that among the homologous genes in Arabidopsis, AtADF5 has the function of polymerizing microfilaments, and AtADF1 has the function of depolymerizing microfilaments^25,29,30^, so it is speculated that AeADF1 may have a microfilament severing function (Fig. S1). To gain insight into the function of the identified plant ADF proteins, we constructed homology models using the SWISS-MODEL server (https://swissmodel.expasy.org/interactive). The intensive mode of SWISS-MODEL uses multi-template modeling for higher accuracy. AeADF1 protein, which showed the highest level of similarity, was selected (Fig. 4A). Multiple sequence alignment of AeADF1 with ADFs from other plant species (*Arabidopsis thaliana*, *Malus domestica*, *Camelina sativa*, *Nicotiana tomentosiformis*, *Rosa chinensis* and *Capsicum annuum*) indicated the presence of a conserved ADF domain in the AeADF1 protein, which is a common characteristic of the ADF family (Fig. 4B). Preliminary studies found that the expression of *AeADF1* in okra is increased under hyperoside treatment. Many literature reports have proposed that the ADF protein plays an important role in the process of microfilament depolymerization^31–34^, suggesting that AeADF1 has the same function. To explore the role of AeADF1 protein in pollen development, the gene expression of *AeADF1* was determined using semiquantitative RT-PCR and quantitative RT-PCR in different flower organs. We used whole flower cDNA as a template to clone *AeADF1* by RACE-PCR. The analysis revealed that *AeADF1* was specifically expressed in the pollen tube. When flowers were sprayed with hyperoside, the transcript level of *AeADF1* increased more in pollen than in petals (Fig. 4C, D).

### AeADF1 colocalizes with F-actin filaments

**Fig. 5:**
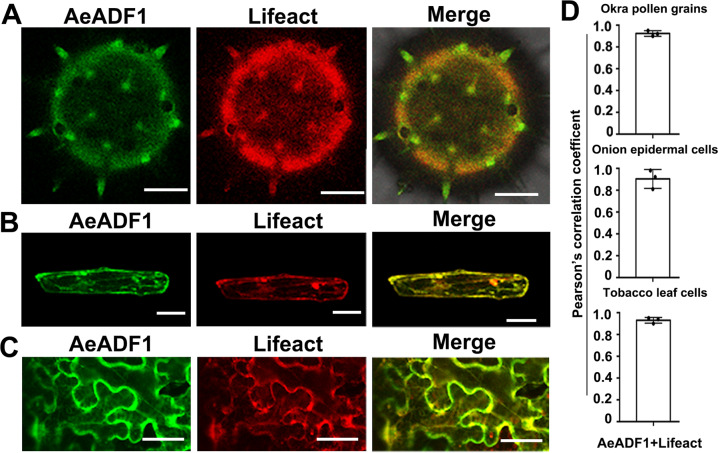
Subcellular localization of AeADF1 and colocalization with F-actin. AeADF1-GFP and the F-actin marker Lifeact-mCherry (Lifeact) were transiently coexpressed in okra pollen grains (**A**), onion epidermal cells (**B**), and *N. benthamiana* leaf cells (**C**). An endoplasmic reticulum marker was used as a control. Scale bars = 50 μm in onion epidermal cells and 2 μm in *N. benthamiana* leaf cells. **D** Pearson’s correlation coefficient of the colocalization of AeADF1-GFP and Lifeact-mCherry in different cells. Each treatment had three biological replicates, and error bars display the standard error of the sample

To examine the ability of AeADF1 protein to act on actin filaments, we first determined whether it colocalizes with F-actin. We used Agrobacterium transient transfection to coexpress *AeADF1-GFP* and *Lifeact-mCherry* in *N. benthamiana* leaves. Particle bombardment was used to transfect the recombinant plasmids of *AeADF1-eGFP* and *Lifeact-mCherry* into onion (*Allium cepa*) epidermal cells and okra pollen. The colocalization of these proteins was observed under a laser confocal microscope. When AeADF1-GFP was expressed together with Lifeact-mCherry in okra pollen, onion epidermal cells, and *N. benthamiana* leaf cells, the signals colocalized with F-actin (Fig. 5A–C). AeADF1-GFP colocalized with free Lifeact-mCherry, indicating that they likely interact with F-actin. Pearson’s coefficient indicated the degree of colocalization in the cell (Fig. 5D). These results suggest that AeADF1 and F-actin were colocalized in pollen and other cells.

### AeADF1 F-actin-severing activity depends on hyperoside

**Fig. 6:**
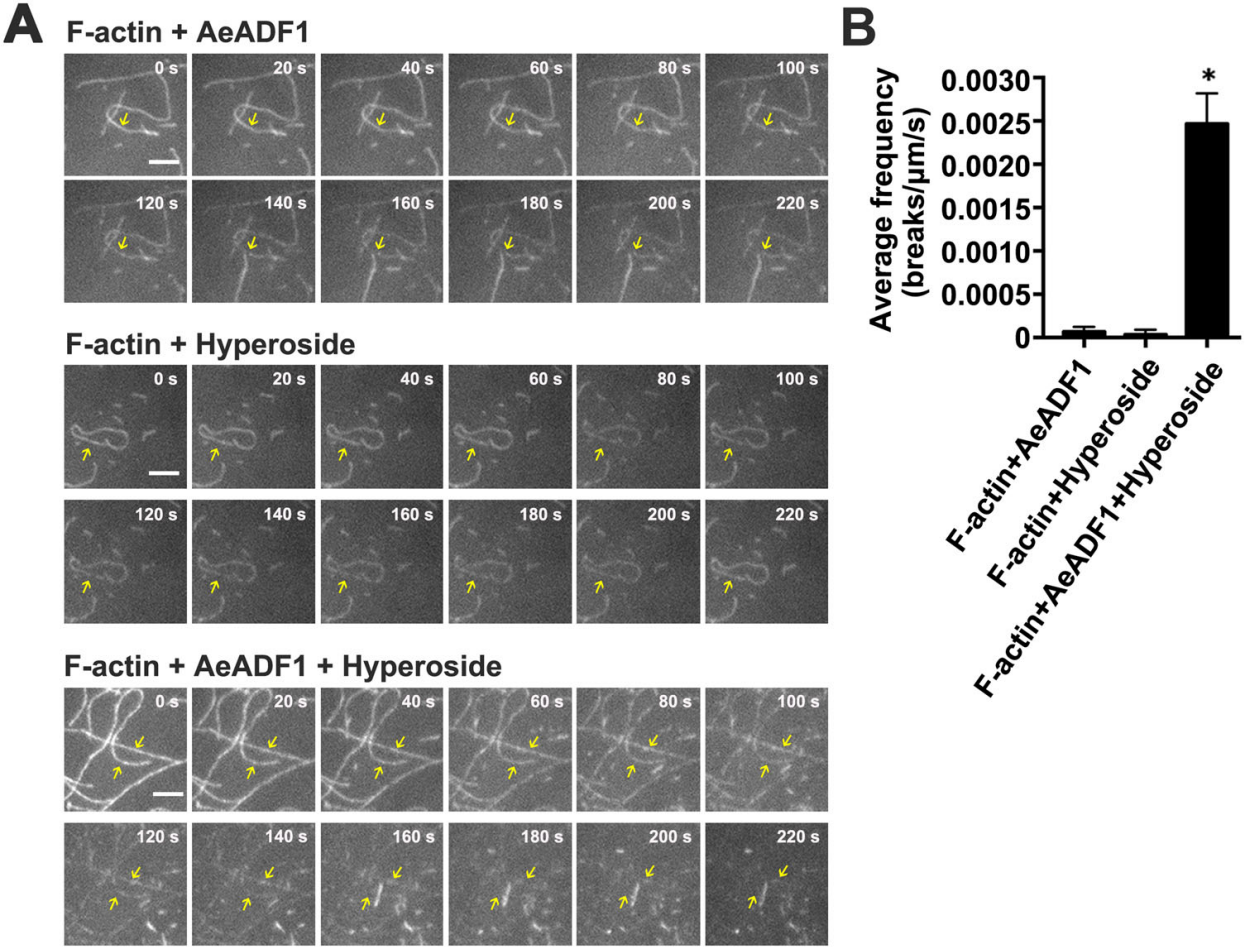
AeADF1 dynamically severs F-actin under hyperoside treatment. **A** After adding 0.1 μM AeADF1, 50 μM hyperoside, or both, prefabricated rhodamine-labeled F-actin was immediately observed through a total internal reflection fluorescence microscope. Bar = 5 μm. **B** F-actin-severing frequencies (breaks μm^−1^ s^−1^) were measured in (**A**). Each treatment had three biological replicates, and error bars display the standard error of the sample. *P < 0.05 (Student’s *t* test)

Based on the above results, we speculated that AeADF1 might play a role in pollen tube growth by regulating F-actin cleavage. To further elucidate the function of AeADF1, we used total internal reflection fluorescence microscopy (TIRFM) to observe the effect of AeADF1 on the cleavage activity of F-actin. We labeled F-actin filaments with rhodamine to facilitate observation. The experimental results showed that with AeADF1 or hyperoside alone, the F-actin filaments were not significantly broken. However, when 50 μM hyperoside and 0.1 μM AeADF1 were added together, the F-actin filaments were broken (Fig. 6A). To show this effect more clearly, we quantified the average frequency of F-actin filament breakage when AeADF1 and hyperoside were added together. The quantitative results showed that compared with adding hyperoside alone, adding hyperoside and AeADF1 together increased the average frequency of F-actin filament breakage by approximately 50-fold (Fig. 6B). The above results all indicate that hyperoside can promote the severing of F-actin by AeADF1.

**Fig. 7:**
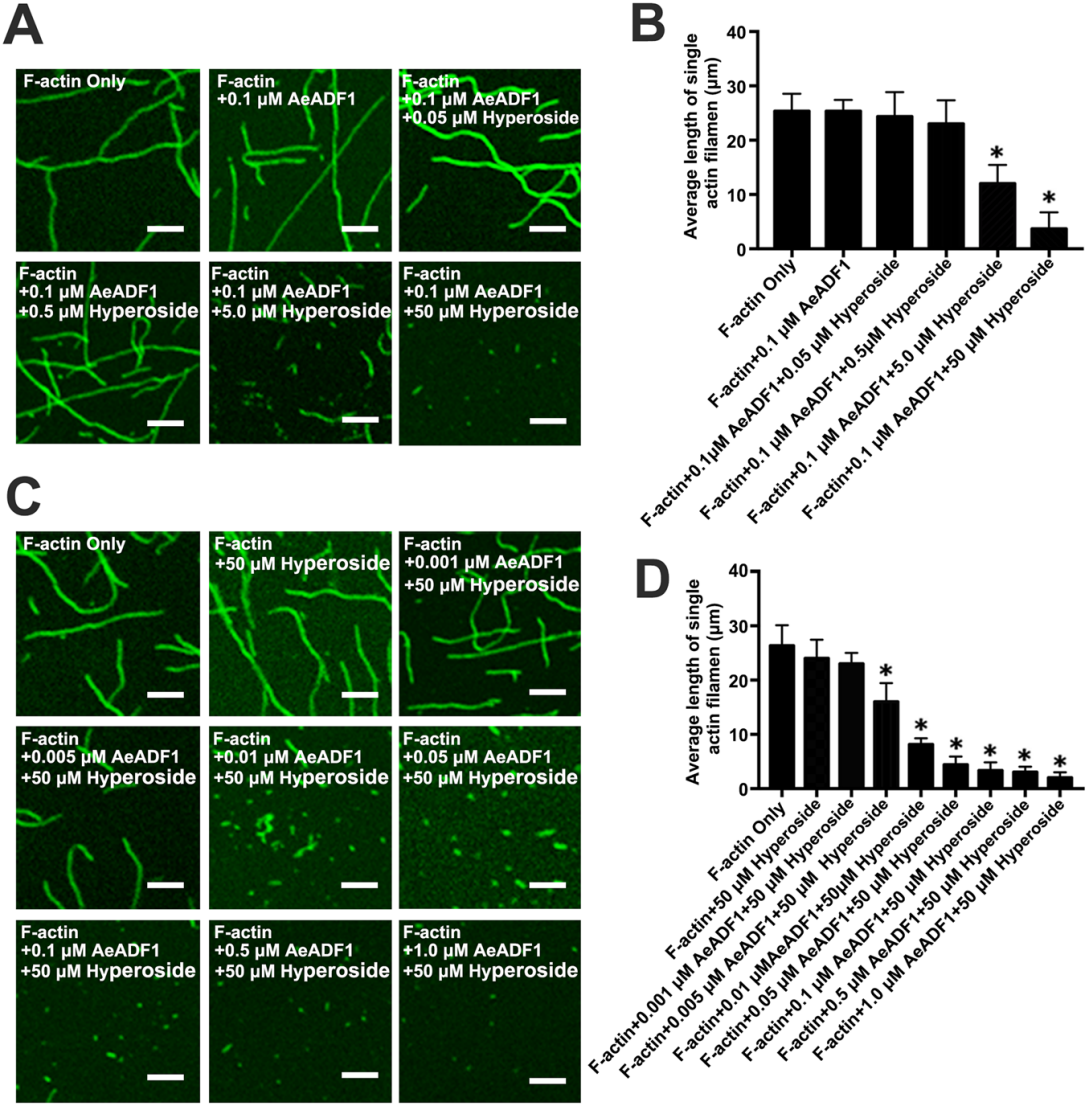
AeADF1 severs F-actin under hyperoside treatment. Actin filaments (polymerized from 0.5 μM G-actin) were visualized by staining with 0.5 μM Alexa-488 phalloidin. **A** F-actin was incubated with 0.1 μM AeADF1 and various concentrations of hyperoside. **B** The average length of individual actin filaments in the reactions from (**A**). **C** F-actin was incubated with 50 μM hyperoside and various concentrations of AeADF1. **D** The average length of individual actin filaments in the reactions from (**C**). In **B**, **D**, each treatment had three biological replicates, and error bars display the standard error of the sample. *P < 0.05 (Student’s *t* test). Scale bar = 5 μm

To test whether AeADF1 is capable of severing F-actin and whether this process is hyperoside-dependent, recombinant AeADF1 protein and polymerized F-actin were incubated for 30 min in the presence of different concentrations of hyperoside. After incubation of purified actin filaments in the presence of free hyperoside, breaks were detected along the F-actin filaments. In the presence of 5.0 μM hyperoside, the average length of F-actin filaments was 11.9 ± 0.12 μm, which was significantly shorter than that of the filaments that formed in the presence of lower concentrations of hyperoside (Fig. 7A, B). At 50 μM hyperoside, we observed a dramatic reduction in the length of the F-actin filaments (Fig. 7A, B). These data showed that in the presence of 0.1 μM recombinant AeADF1, filament length decreased as the concentration of hyperoside increased, proving that the number of breaks substantially increased with increasing hyperoside concentration. To further analyze whether AeADF1 severing of F-actin depends on its concentration in the presence of a high concentration of 50 μM hyperoside, we incubated various concentrations of AeADF1 (0.001–1.0 μM) with preformed F-actin for 30 min. In the presence of 0, 0.001, 0.005, 0.01, 0.05, 0.1, 0.5, and 1.0 μM AeADF1 and 50 μM hyperoside, the average lengths of the F-actin filaments were 27.1 ± 0.24, 25.5 ± 0.05, 23.1 ± 0.07, 17.9 ± 0.04, 9.2 ± 0.05, 2.3 ± 0.05, 2.2 ± 0.07, and 2.1 ± 0.02 μm, respectively. Thus, the average filament length decreased sharply when the AeADF1 concentration was greater than 0.01 μM but then leveled off.

### The inhibitory expression of *AeADF1* in pollen tubes reduces the promotion of pollen tube growth by hyperoside

**Fig. 8:**
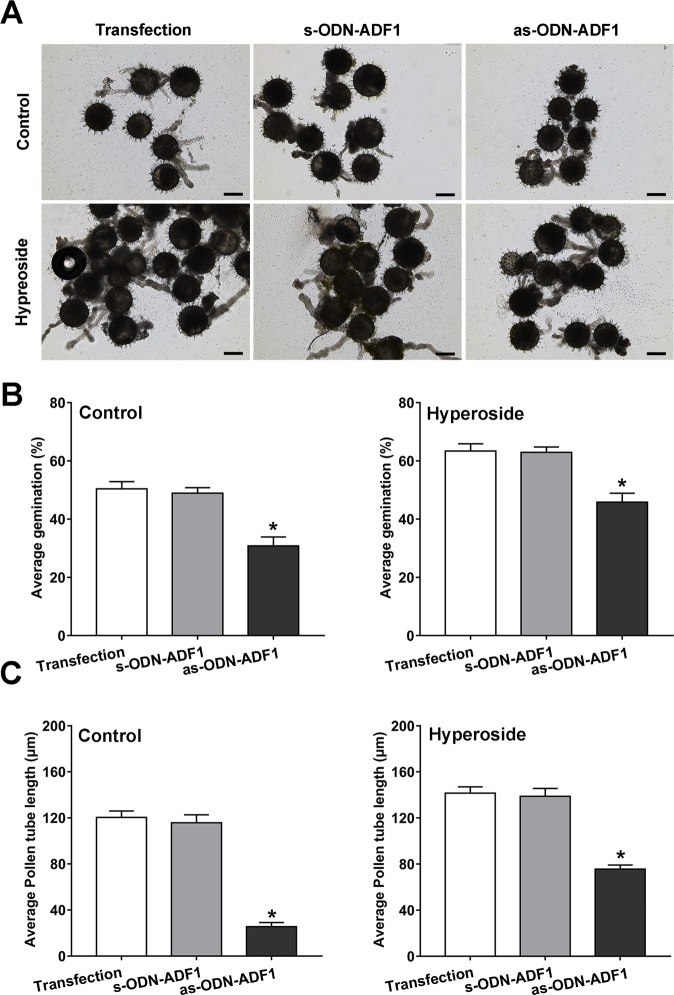
Decreased gene expression of *AeADF1* leads to the inhibition of pollen germination and pollen tube growth. **A** The germination status of oligonucleotide-transfected pollen tubes in the control and treatment with hyperoside (0.1 mM) groups. Scale bar = 80 μm. **B**, **C** The length of the pollen tube and germination rate when treated or not treated with hyperoside for 40 min. Each treatment had three biological replicates, and error bars display the standard error of the sample. *P < 0.05 (Student’s *t* test)

To further prove the role of *AeADF1* in pollen germination and pollen tube growth, we used oligonucleotide technology to inhibit the expression of *AeADF1* in pollen and observed its pollen germination rate and pollen tube growth in the control and hyperoside treatments for 40 min. Compared with the s-ODN-ADF1 and the control groups, the expression of *AeADF1* was significantly reduced in the as-ODN-ADF1 group (Fig. S2). In the control, the inhibitory expression of *AeADF1* reduced the pollen germination rate and pollen tube length. The application of exogenous hyperoside can partially recover the pollen germination rate and pollen tube length. The above results showed that hyperoside can promote the expression of *AeADF1* to have a positive effect on pollen germination and pollen tube growth (Fig. 8).

## Discussion

**Fig. 9:**
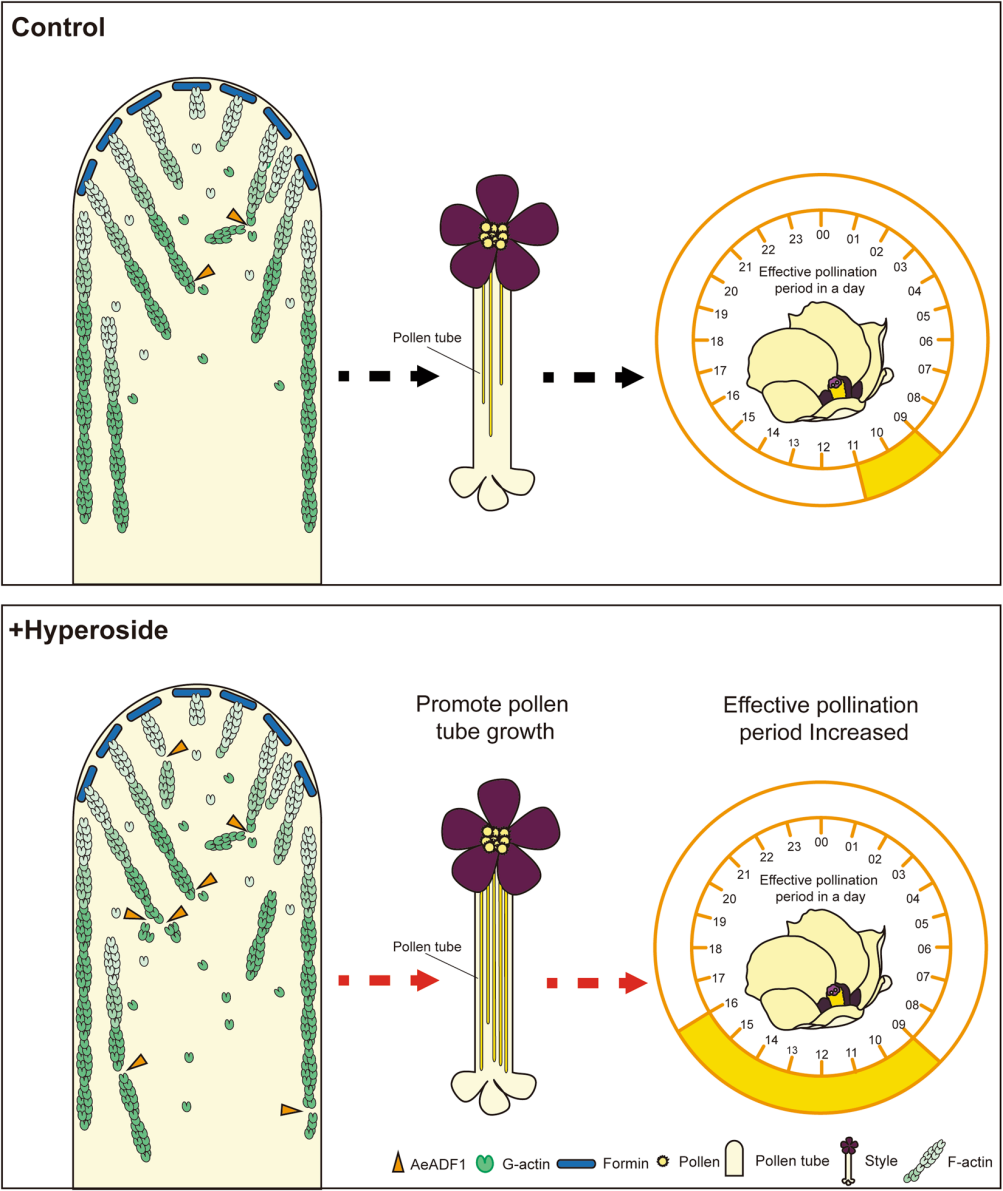
A proposed model of the role of *AeADF1* in the pollen tube. The exogenous application of hyperoside increases the expression of *AeADF1* in the pollen tube, thereby enhancing its microfilament severing ability. Finally, it promotes pollen germination and pollen tube growth, prolonging the effective pollination period. The red arrow indicates that this part of the effect has been enhanced

Flavonoids are secondary metabolites that widely accumulate in plants and are present in various plant tissues^35^. Previously, most studies on the role of flavonoids in flower development at home and abroad focused on the accumulation of secondary metabolites of flavonoids playing a major role in determining the color of plants, especially anthocyanins^36–39^. Anthocyanins increase the attractiveness of plants to pollinators, resist ultraviolet rays, and defend against pathogens^40^. For example, the three proteins MYB, bHLH, and WDR can interact and form the MBW complex to promote the accumulation of anthocyanins in plants. This phenomenon has been reported in petunia, grape, and poplar^41,42^. Our previous research proved that flavonoids are involved in the flower development of *A. esculentus* and play an important role as signaling substances. Exogenous application of hyperoside promotes the synthesis of hyperoside in okra, thereby promoting okra’s fruit set rate^43^. This study proves that the exogenous application of hyperoside prolongs the effective pollination period of okra, promotes the expression of the *AeADF1* gene, and promotes the depolymerization of AeADF1 on microfilaments. The exogenous application of hyperoside can promote pollen germination and pollen tube growth in okra. The oligonucleotide transfection experiment of *AeADF1* gene proved that the inhibitory expression of *AeADF1* reduced the rate of pollen germination and inhibited the growth of pollen tubes. Exogenous application of hyperoside to pollen with inhibited expression of *AeADF1* can partially alleviate this inhibition. This shows that *AeADF1* plays an important role in pollen germination and pollen tube growth in *A. esculentus*, and hyperoside has a promoting effect on AeADF1 protein. This research laid a molecular foundation for analyzing the effect of flavonoid signal transmission on protein activity and protein modification in other plants (Fig. 9).

The role of the actin cytoskeleton in pollen germination and pollen tube growth is essential. As a kind of actin-binding protein, ADF proteins are abundant in eukaryotic cells. They play a significant role in maintaining the dynamics of actin. In this process, the homologous region (long alpha helix) of ADF can depolymerize F-actin by enhancing the ability of ADF to bind to actin. An important function of ADF depolymerization of microfilaments is to form a new open end, which is usually considered to be the cause of the formation of a microfilament network^44–46^. Studies have reported that in *A. thaliana*, the shortening of cell filament bundles is due to the overexpression of actin depolymerization factor 1 (*ADF1*). AtADF1 can bind to actin and promote the depolymerization of microfilaments in vitro. The depolymerization ability increases with increasing AtADF1 concentration. The decreased expression of *AtADF1* in the adf1 mutant leads to an increase in actin bundles, which in turn reduces flowering time. In this study, we speculate that *AeADF1* has a similar function to *AtADF1* in Arabidopsis. Both proteins can directly cleave F-actin, and high concentrations of ADF protein have a higher ability to cleave F-actin^47–49^. At the same time, the depolymerization of microfilaments can promote pollen germination and pollen tube growth. In our previous research, *AeADF5* may also play a significant role in pollen tube growth^43^. Based on the functions of *ADF5* homologs in other plants, we speculate that the protein has the function of polymerizing actin. The verification of the function of *AeADF5* in *A. esculentus* and the synergy of *AeADF5* and *AeADF1* need further elucidation.

In flowering plants, wind and animals distribute pollen in different environments, driving the spread of genetic variation within a species. Successful fertilization of flowers in unpredictable climates is a key factor in determining plant yields^50,51^. Under proper conditions, to complete fertilization, mature pollen needs to germinate on the stigma and then extend its pollen tube to deliver sperm cells to the ovule. The factors that control the successful germination of pollen have always been the focus of plant reproduction, evolution, and breeding research^52,53^. With continuous research, we now have a certain understanding of the regulatory mechanisms that control the fertilization process, such as signal transduction pathways and cytoskeletal proteins. However, most research has focused on model plants. Our related research in a non-model plant, *A. esculentus*, proved the regulatory effect of the ADF1 protein on pollen germination and pollen tube growth. At the same time, it was found that the hyperoside content increased significantly during flowering. As a flavonoid, hyperoside has many physiological properties, such as anti-inflammatory, antispasmodic, diuretic, and antitussive properties, and can lower blood pressure, lower cholesterol, and protect the heart and cerebral blood vessels; thus, it is an important natural product. The development of new varieties of okra with increased hyperoside content has an important impact on the medical and economic benefits of the plant^54–56^. This research lays a molecular foundation for the development of fine varieties of okra and provides a new research direction related to flower development in other non-model plants.

## Materials and methods

### Plant materials

*A. esculentus* seeds were sown in the greenhouse in April, and they were transplanted to the field after 20 days of growth. Plants began to bloom in early July, and blooming dynamics were recorded beginning on July 15th. Blooming flowers, pollen, and other tissues were collected and stored at −80 °C until use.

### Application of hyperoside solution

As reported by Yang et al.^6^, we configured the stock solutions. When applied externally, 1 liter of the solution was sprayed for every ten plants. The average height of the sprayed plants was 1 m, and the average canopy width of the sprayed plants was 50 cm. The okras were sprayed every 2 days for a total of four times, and the control was sprayed with buffer only.

When hyperoside was used to treat *N. benthamiana* leaves, a 0.1 mM solution of hyperoside was first prepared. Then, 1 cm *N. benthamiana* of cut leaves were soaked in hyperoside solution for 5 min and then observed under a microscope.

### Effective pollination period and pollen tube growth assay

As a result of previous research findings, it takes ~24 h for okra to transition from flowering to withering^6^. Therefore, we determined the effective pollination period as the ratio of the time spent on flowers in 24 h.

As reported by Meng et al.^57^, the pistils after pollination were collected and fixed in phosphate-buffered saline. When staining with aniline blue, the fixed pistil was rinsed with running distilled water three times and then placed in 1 M NaOH for 12–16 h to soften it. After softening, the sample was rinsed five times with distilled water. Finally, the pistil was put into a 0.1% aniline blue solution and placed in the dark for 12–16 h. After removal, the pollen tube was observed under a confocal microscope (Leica SP8). Then, the average length of the pollen tube in the pistil was measured. We used the average length of the pollen tube in the pistil as the pollen tube growth indicator.

### Pollen germination rate and pollen tube length analysis

Fresh okra pollen was picked and placed in okra pollen germination solution for 2 h at 25 °C in the dark. Then, the pollen germination rate and pollen tube length were observed under a microscope. Each treatment had three biological replicates, and 20 visual fields were selected for each replicate under a tenfold microscope for statistical analysis.

### RNA isolation and qRT-PCR analysis

As reported by Meng et al.^58^, the CTAB method was used for sample RNA extraction. After removing DNA contamination with RQ1 DNase (Promega, WI, USA), an RT reagent kit (Takara) was used to reverse transcribe 1 μg of total RNA into cDNA. Fast SYBR Mixture (CWBIO, Beijing, China) was used in an Icycler iQ5 (BioRad, CA, USA) instrument to perform qRT-PCR experiments according to the corresponding instructions. When qRT-PCR was executed, there were three technical replicates for each sample, and the expression of housekeeping genes was used as the internal reference for sample standardization. The transcription level was calculated using the 2^−∆∆Ct^ method. The qRT-PCR primers of all genes are shown in Supplemental Table 1.

### Bioinformatics analysis of AeADF1

AeADF1 and other candidate protein sequences were queried against the SMART database (http://smart.embl-heidelberg.de/, accessed October 10, 2016) to test whether they were predicted to be members of the ADF family. Structural motif annotation was performed using DNAMAN7 software. SWISS-MODEL (https://swissmodel.expasy.org) was used for three-dimensional structure prediction.

### Subcellular colocalization with F-actin

*AeADF1* and *eGFP* were fused and inserted into the p-CAMBIA1300 vector controlled by the 35S promoter. The Lifeact-mCherry fusion protein was used as an F-actin marker. Constructs were transformed into *Agrobacterium tumefaciens* strain GV3101. As mentioned above, the Agrobacterium liquid was centrifuged to remove the supernatant, and the pellet was resuspended in buffer^59^. Using a 5 mL syringe, the resuspension solution was injected into the leaves of *N. benthamiana* and then incubated in a greenhouse at 22–24 °C for 72 h. The *N. benthamiana* leaves were collected, and protein fluorescence was observed under a fluorescence microscope.

*AeADF1-eGFP* and *Lifeact-mCherry* were added to the pEZS-NL vector. Then, the vector plasmid was evenly mixed with spermidine, 2.5 M calcium chloride, and a standard concentration of gold for use. Next, particle bombardment was used to transfect the recombinant plasmid into onion epidermal cells, and the bombarded onion epidermis was cultured in the dark for 12–18 h. Then, the onion cells were placed under a laser confocal microscope to observe the subcellular localization of genes, as described by Meng et al.^60^.

We calculated the Pearson correlation coefficient in the cell according to the method described by Yang et al.^61^. We used the Pearson correlation coefficient as an indicator of the linear correlation of eGFP and mCherry fluorescence intensity. First, ImageJ software was used to convert the eGFP fluorescence value in the cell to a gray value and define it as *X*, and the gray value was converted from the mCherry fluorescence value as *Y*. The expected values of *X* and *Y* were defined as *E*(*x*) and *E*(*y*), respectively, and the covariance was derived from them. Next, the standard deviation of the *X* variable (*rX*) and *Y* variable (*rY*) was calculated, and the final Pearson coefficient was calculated as Cov/*rX*, *rY*. The closer the value is to 1, the greater the colocalization of eGFP and mCherry.

### Total internal reflection fluorescence microscopy assay

As described by Yang et al.^61^, the severing of F-actin was visualized. Before use, G-actin was labeled with rhodamine and then centrifuged for 2 h. Then, F-actin was added to the flow cell and incubated for 5 min, and the combination of AeADF1 and hyperoside was added to each flow cell. The 1003/1.45 oil objective lens was used immediately to observe the severing of F-actin with a rotating disk confocal microscope. Images were captured and collected by a TIRFM. Images were acquired every 2 s for 220 s, and all images were analyzed with ImageJ.

### Fluorescence microscopy assay

As previously described by Zhou et al.^62^, imaging of F-actin was performed in vitro. First, all proteins were centrifuged at 50,000 × *g* for 30 min. F-actin (0.5 μM) was incubated with 0.5 μM AeADF1 at room temperature for 10 min and then fixed with 1% glutaraldehyde. Aliquots (1 μL) of the samples were placed onto a slide and observed using a confocal microscope.

### Protein purification

*AeADF1* was added to a vector with a His-tag and transformed into *Escherichia* *coli* BL21. Isopropyl-b-D-thiogalactopyranoside (1 mM) induced the growth of *E. coli* BL21 cells, which were grown at 16 °C for 16 h. A chromatographic column containing 2 mL of Ni-NTA Sepharose was used to elute and purify the AeADF1 protein from cell lysates.

Fresh rabbit muscle tissue was removed, and the tendon was buried in ice for preservation. After the muscle was minced, it was extracted with KCl, EDTA, double-distilled H_2_O, and acetone in sequence and then subjected to a series of precipitation and dialysis reactions to obtain actin protein.

### Antisense oligonucleotide transfection

As mentioned before^60^, phosphorothioate antisense oligodeoxynucleotides (as-ODN) and sense oligodeoxynucleotides (s-ODN) for AeADF1 were designed, and s-ODN was used for comparison. Here, 10 mM as-ODN, s-ODN, and transfection agent were added to the pollen grain germination solution for 40 min, and the pollen germination rate and pollen tube length were determined.

## Supplementary information

Supplemental Table

Supplemental Figure

Without treatment in the leaves of N. benthamiana transiently expressing Lifeact-GFP in 10 min

Treatment with buffer solution in the leaves of N. benthamiana transiently expressing Lifeact-GFP in 10 min

Treatment with hyperoside (0.1 mM) in the leaves of N. benthamiana transiently expressing Lifeact-GFP in 10 min
