# Supplementary material for: Hyperoside promotes pollen tube growth by regulating the depolymerization effect of actin-depolymerizing factor 1 on microfilaments in okra
Source: Hortic Res. 2021 Jul 1;8:145. doi: 10.1038/s41438-021-00578-z (PMC8245483; doi:10.1038/s41438-021-00578-z)
Supplement: Supplementary file 1 — Supplemental Table [file 41438_2021_578_MOESM1_ESM.docx]

Table S1. Primer sequences list for qRT–PCR analyses

| **Gene name** | **Primer（5'-3'）** |
| --- | --- |
| house-keeping gene-F | ATTTGCCAGCAGACCTTCTACAAT |
| house-keeping gene-R | TACTTACTGTCTGGAGGAGCAACAA |
| AeADF1-F | AGTTCGCTGCTAGTTTACCTGCTG |
| AeADF1-R | GCAATGTCAGGACACCAAGCAATG |
| AeADF2-F | GGCTGCTGCTGCTTCTGGTATG |
| AeADF2-R | TCTCTATCTTGCGGCGGATTGTTC |
| AeADF3-F | GGGGCAAACCGTTGAAAACTTAGC |
| AeADF3-R | GGAGCACCTTCTGACGACACAAAG |
| AeADF4-F | TGAGAACTTTGCGGCGTCTTTACC |
| AeADF4-R | TTCGCAACGTTAGGACACCAAGC |
| AeADF5-F | TGCGGATTCTCTTGCGGTTGAC |
| AeADF5-R | GGGCTCCACGCAATCCTGAAC |
| AeADF6-F | TTGTGCTGCTTGGTCACCTTCC |
| AeADF6-R | AGGTTCACTTCGGTCTGGATCTCG |
