# Supplementary material for: Hyperoside promotes pollen tube growth by regulating the depolymerization effect of actin-depolymerizing factor 1 on microfilaments in okra
Source: Hortic Res. 2021 Jul 1;8:145. doi: 10.1038/s41438-021-00578-z (PMC8245483; doi:10.1038/s41438-021-00578-z)
Supplement: Supplementary file 2 — Supplemental Figure [file 41438_2021_578_MOESM2_ESM.docx]

**
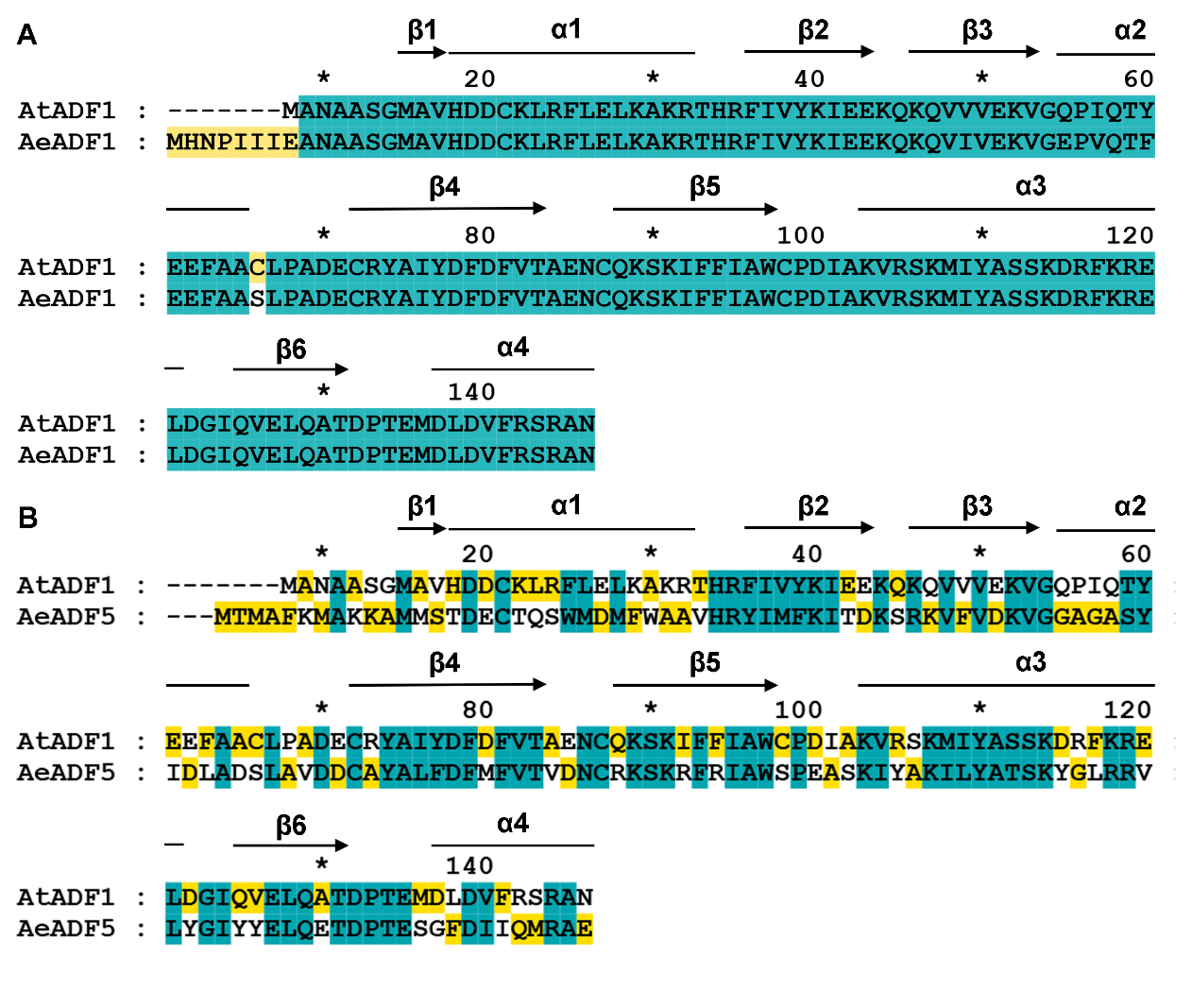
**

**Figure S1. The protein sequences of AeADF1 and AeADF5 were aligned with AtADF1 respectively.**

The α-helix sequence in AtADF1 is marked with a straight line, and the β-sheet sequence in AtADF1 is marked with an arrow.

**
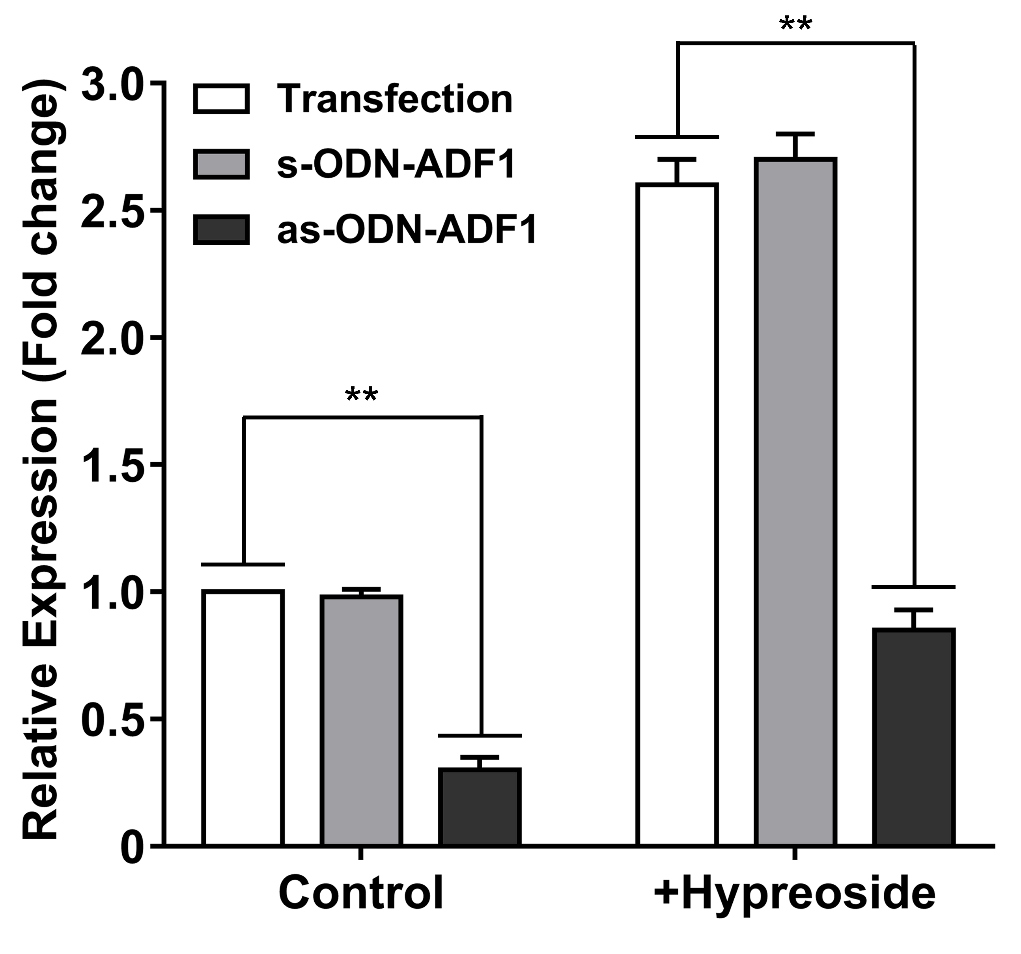
**

**Figure S2. The gene expression of *AeADF1* in transfection, s-ODN-ADF1 and as-ODN-ADF1 Lines with hypreoside treatment or not.**

The mean and SE from three independent experiments are shown. ** indicates significant differences in comparison with the transfection lines in control at p<0.01.
